# Concurrent validity of an isokinetic lift test used for admission to the Swedish Armed Forces

**DOI:** 10.1371/journal.pone.0207054

**Published:** 2018-11-06

**Authors:** Tony Bohman, Matthias Tegern, Alexandra Halvarsson, Lisbet Broman, Helena Larsson

**Affiliations:** 1 Department of Neurobiology, Care Sciences and Society, Division of Physiotherapy, Karolinska Institutet, Stockholm, Sweden; 2 Department of Community Medicine and Rehabilitation, Umeå University, Umeå, Sweden; 3 Allied Health Professionals Function, Karolinska University Hospital, Stockholm, Sweden; 4 Swedish Armed Forces, Headquarters, Medical Services, Stockholm, Sweden; Sao Paulo State University - UNESP, BRAZIL

## Abstract

The aim of this study was to assess the concurrent validity of the IsoKai isokinetic lift test peak force (IsoKai_Peak_) in comparison to a submaximal 5-10RM deadlift test (5-10RM_DL_), and to develop an equation for converting the IsoKai_Peak_ in Newton (N) to an estimated 1RM (1RM_est_) deadlift load in kilograms (kg). The participants included 28 males and 16 female employees in the Swedish Armed Forces (20–59 years). Each participant conducted the IsoKai lift test, followed by the 5-10RM_DL_ test at one occasion. The Pearson′s correlation coefficient, with a 95% confidence interval was calculated to evaluate the validity between the IsoKai_Peak_ and the 1RM_est_ deadlift load derived from the 5-10RM_DL_ test. Univariate and multivariable linear regressions were used to derive the equation for calculating the 1RM_est_ deadlift load based on the IsoKai_Peak_. The IsoKai_Peak_ showed *good- to-excellent* correlation with the 1RM_est_ deadlift weight with a correlation coefficient of 0.84 (0.72–0.91) for the total sample, and 0.65 (0.37–0.83) and 0.81 (0.53–0.93) in males and females, respectively. The final equation, 1RM_est_ deadlift weight (kg) = -51.63 + (0.08 x IsoKai_Peak_) + (2.28 x BMI), explained 72% (adjusted R^2^ = 0.72) of the total variance in the 1RM_est_, and had a standard error of the estimate (SEE) of 16.57 kg. In conclusion, the IsoKai isokinetic lift test could be considered a highly valid measure of maximal dynamic muscular strength in comparison to the 5-10RM_DL_. The equation can be used to convert the IsoKai lift test (N) results to an 1RM_est_ deadlift load (kg), but with consideration of the relative large SEE.

## Introduction

Measures of muscular strength are of importance in health care, sports and sports medicine, and in physically demanding occupations, e.g. soldiers, firefighters and police officers, where strength tests are commonly used for job selection and assessment during employment [1–5].

There are several ways to measure dynamic muscular strength. However, the one repetition maximum (1RM) test, identifying the maximum weight a person can move once throughout the full range of a movement in a controlled manner, is regarded as the “standard” [2, 6]. The 1RM test has proven to be highly reliable (test-retest reliability coefficients; 0.74–0.99) [6]. An approach commonly considered safer to establishing the 1RM is to perform a submaximal repetition maximum test with repetitions to failure [2, 6, 7]. The general recommendation is to use no more than 10RM in a submaximal RM test in order to accurately estimate the 1RM, where 5-10RM are a commonly used estimation method [2, 7, 8].

Another alternative to assess dynamic muscular strength is to use an isokinetic strength test, which involves assessment of strength during a movement performed at constant speed and with resistance proportional to the amount of force produced during movement [1]. Isokinetic tests are considered to be highly reliable, fast and easy to assess, and relatively safe since the resistance adapts to muscle force produced [2, 6].

**Fig 1:**
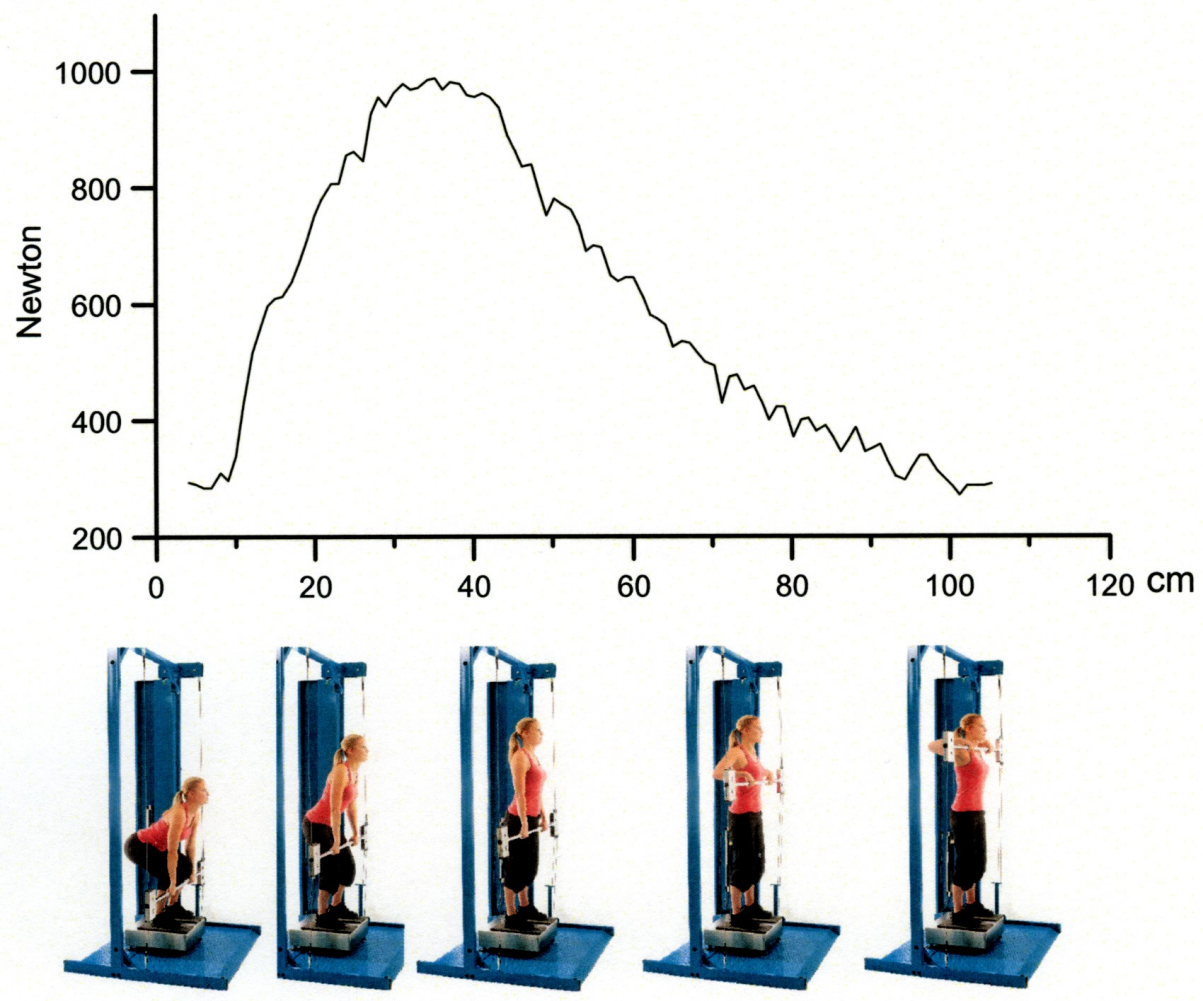
An IsoKai isokinetic lift test and the corresponding force curve expressed as Newton per cm of the lift.

The IsoKai isokinetic lift test has for several years been used to assess dynamic muscular strength on admission to service in the Swedish Armed Forces (SwAF) and the Swedish Police [9]. The IsoKai lift test is performed in a mechanic device during a maximal two-handed lift of a weight-lifting bar from “floor” to shoulder level (Fig 1).

The peak force value (IsoKai_Peak_, in Newtons), which represents the maximum dynamic muscle force produced during the lift, is registered when the bar reaches about hip level. The lift from “floor” to hip level mimics “deadlift”, a common free weight lift used in strength training.

The IsoKai isokinetic lift shows excellent content validity (content validity index > 0.78) with soldier’s tasks of lifting, carrying with hands and digging [10]. This association with military tasks performance is critical. However, the IsoKai lift (IsoKai_lift_) test has not yet been assessed for its validity to the underlying criterion of muscle strength [11]. A drawback with the IsoKai_lift_ test is that the measure of strength is produced during constant speed and varying load, and not during constant load and varying speed as is done during lifting in real life. Because dynamic muscle strength is commonly measured in weight, e.g. 1RM in kg, an accurate way of converting the IsoKai_lift_ test measure to a 1RM lifting capacity in kg would be of practical use in admission and other job selection procedures, as well as helpful in determining the intensity of strength training.

The aims of this study were (1) to assess the concurrent validity of the IsoKai_lift_ test, as administered during admission to the SwAF, in comparison to a submaximal 5-10RM deadlift test (5-10RM_DL_), and (2) to develop an equation for converting the peak force produced from the IsoKai_lift_ test (IsoKai_Peak_) in Newton to an estimated 1RM (1RM_est_) deadlift weight in kg.

## Material and methods

### Design and study sample

Participants in this validity study comprised voluntary employees from the SwAF. To be included, participants had to be without any pathology that could pose a risk of injury, or influence the test performance. Participants received written information and signed a written informed consent. Prior to testing, participants had to fill in a questionnaire concerning personal data, employment, physical workload and leisure physical activity (S1 Appendix) [12]. The study was approved by the Regional Ethic Committee of Stockholm, Sweden, dnr 2016/2073-32. Characteristics of the participants are presented in Table 1.

**Table 1:** Characteristics of the study sample.

	All *(n = 44)*	Males *(n = 28)*	Females *(n = 16)*
Age (years), median (IQR)	33 (14)	34 (16)	30 (10)
Height (cm), mean (SD)	175 (9)	180 (6)	168 (7)
Weight (kg), mean (SD)	78 (12)	83 (9)	68 (10)
BMI (kg/m^2^), mean (SD)	25 (3)	26 (2)	24 (3)
Leisure physical activity, n (%)			
Low	1 (2)	1 (4)	0
Moderate	0	0	0
High	13 (30)	7 (25)	6 (38)
Very high	30 (68)	20 (71)	10 (62)
Physical workload, n (%)			
Sedentary	34 (77)	21 (75)	13 (81)
Low	8 (18)	5 (18)	3 (19)
Moderate	2 (5)	2 (7)	0
High	0	0	0

IQR; interquartile range, SD; standard deviation, BMI; body mass index.

### Test procedure

The tests were conducted at the Swedish Defence Recruitment Agency in Stockholm, Sweden, in 2017. The IsoKai_lift_ were administered by two licensed nurses who have several years of working experience with the IsoKai_lift_ test. The submaximal 5-10RM_DL_ tests were overseen by a registered physiotherapist (TB) who is well-experienced in muscular strength testing. Both tests were performed on the same day, and the testing procedure followed a standardised test protocol. Study participants were informed of the test procedure verbally and by watching a short video clip containing the tests. A ten-minute warm-up session using a calibrated cycle ergometer with 1.5 kp resistance and a pedalling rate of 60 rpm (= 100 Watts), was followed by the IsoKai_lift_ test, a ten-minute resting period, and the 5-10RM_DL_ test. The IsoKai_lift_ test was regarded as the least stressful strength test and for that reason it was performed prior to the 5-10RM_DL_ test. Participants were blinded to the test results during the procedure. Both tests were performed with the test administrator and the participant being alone in a room. Further, the test administrators were not allowed to verbally encourage the participant during testing. Participants could terminate all tests at any time and were told to stop if they experienced any pain or discomfort during testing.

### Assessments

#### The IsoKai isokinetic lift (IsoKai_lift_) test

The IsoKai_lift_ measures muscular strength during a vertical lift from about 0.3 m from the floor to shoulder level. The device consists of a frame supporting an international free-weight lifting barbell, connected to a hydraulic system by two wires that regulate the speed of the lift at 0.3 m/sec. For each cm of lift produced, the muscular force (N) is registered by a force plate. The length of the test participant is measured by an electronic instrument connected to the device, and weight is registered by the force plate. Prior to testing, the administrator practically demonstrated the lift to secure a safe testing procedure, and the participant was allowed to perform one submaximal test to get used to the technique. Then, the participant conducted the test lifts, standing with feet separated by shoulder width, lifting the barbell using a double overhand grip as, fast as possible (Fig 1). The results were registered as the mean force value (N) of the lift and the peak force value (N), representing the maximal force produced during the lift (Fig 1). Each participant performed three to four lifts with at least two-minute rest in between, of which the highest IsoKai peak force value (IsoKai_Peak_) was used in further analyses.

#### The 5-10RM deadlift (5-10RM_DL_) test

Each participant conducted a 5-10RM_DL_ test using an international free-weight lifting barbell of 20 kg and weight plates ranging from 2.5 kg to 25 kg [2, 7]. The test performance was standardised according to recommendations from the American College Sports Medicine [8]. The speed of the lift followed a metronome set at 90 beats per minute, with one lift completed within 4 beats. The test administrator demonstrated the lift using the technique suggested by Farley [13]. Next, the participants familiarised themselves with the technique using a wooden stick, followed by a five repetition deadlift test with a load of approximately 50% of their body weight. The test load was individually chosen with regards to the participant’s body weight, physical activity level, training experience and subjective evaluation of the testing load, using the Borg rating of perceived exertion scale [14]. After a short rest, the 5-10RM_DL_ test was executed. If the test resulted in less than 5, or more than 10, repetitions, or if the participant were unable to keep the speed or the correct technique, a short rest was followed by a second testing with weights adjusted if needed. A maximum of three tests were allowed.

#### Calculating 1RM_est_ from 5-10RM_DL_ test results

To calculate the 1RM_est_ deadlift weight from the 5-10RM_DL_ test, we used the equation developed by Mayhew et al.; 1RM = 100 x rep weight / (52.2 + 41,9 x exp [-0.55 x reps]) [15]. In this equation, *rep weight* stands for the test weight, *reps* the number of repetitions used, and *exp [x]* the natural logarithm of x to the base of the mathematical constant “e”. The equation has good accuracy for males as well as for females, and has been cross-validated in several populations [15, 16]. According to LeSure et al., the equation underestimates 1RM by about 10% when deadlift is used as test procedure. Hence, a multiplicative factor of 1.10 was added to the original equation [17].

### Statistics

Descriptive data were presented as means and standard deviations (SD) or medians and interquartile range (IQR). Concurrent validity was assessed using the Pearson′s correlation coefficient (*r*), and the 95% confidence intervals (95% CI) were calculated using the Fisher′s transformation recommended for sample sizes less than 100 [18]. The strength of the association was evaluated with a correlation ranging from 0.50 to 0.75 indicating a *moderate-to-good* association, and a correlation above 0.75 indicating a *good-to-excellent* association [11]. The coefficient of determination (R^2^) was utilised as the percentage of variance in the calculated 1RM_est_ that can be explained by the IsoKai_Peak_ [11, 19]. A scatterplot was used to visualise the correlation between measures, and the paired t-test was used to assess the potential differences between the measures. To facilitate comparison between the measures, the IsoKai_Peak_ was converted from N to kg (1 N = 0.102 kg) in the analyses.

An ‘a priori’ sample size calculation showed that 38 participants was needed to detect a correlation of at least 0.5 with an alpha level of 0.05 and statistical power of 0.90.

#### Development of the equation to convert IsoKai_Peak_ to a 1RM_est_

The development of the equation for determining the 1RM_est_ deadlift weight (kg) by the IsoKai_Peak_ (N) was done in two steps using linear regression; univariate analyses followed by a multivariable backward deletion procedure [19].

In the univariate analyses, the following factors that could potentially be associated to the 1RM_est_ were considered: sex, weight, height, BMI, leisure physical activity and physical workload. A univariate linear regression was performed for each of the factors. Factors with a p-value < 0.2 (Wald test), for the association with the 1RM_est_, were considered as candidate factors for the multivariable analyses [19]. The associations between the candidate factors were tested for collinearity, which was judged to be present if the Spearman′s correlation between numeric variables was greater than 0.5, or when significant associations were found between categorical variables (Chi-square test) or between categorical and numeric variables (Kruskall-Wallis test). The presence of collinearity was managed by eliminating, from the analyses, factors that were judged to be the least important from a logical perspective.

In the multivariable analysis, a sequential backward manual selection procedure based on linear regression was performed, including the candidate factors from the univariate analyses [19]. The factor with the highest p-value (Wald test) was excluded one by one until all factors in the model had a p-value < 0.1 [19]. The association between the included factor and the outcome was reported as beta-coefficient (β) with standard error (SE) and corresponding 95% CI, and standardised beta-coefficients (B). The accuracy of the equations was established using the adjusted R^2^ and the standard error of the estimate (SEE) [11, 19]. Assumptions of linearity and homoscedasticity were utilised using residual plots and restricted cubic splines [19].

Statistical analyses were performed using Stata/IC version 14.2 (StataCorp LLC, USA). The level of significance was set to 95%. Graphics were constructed using IBM SPSS Statistics version 23 (IBM Corporation, USA).

## Results

All 44 participants completed the tests. Twenty participants needed four attempts with the IsoKai_lift_ test, whereas nine needed two attempts with the 5-10RM_DL_ test to obtain valid results. No adverse events occurred during the testing procedures.

The result showed a good-to-excellent association between the IsoKai_Peak_ and 1RM_est_ deadlift weights in the total sample, as well as among males and females (Table 2). The IsoKai_Peak_ explained 70% (R^2^; 0.70) of the total variance in the 1RM_est_ weight in the total sample.

**Table 2:** Correlation and coefficient of determination for the association between the IsoKai_Peak_ (kg) and the estimated 1RM (1RM_est_) weights (kg) derived from 5-10RM deadlift (5-10RM_DL_) tests.

	*r*	95% CI	R^2^
All (*n = 44*)	0.84	0.72–0.91	0.70
Males (*n = 28*)	0.65	0.37–0.83	0.43
Females (*n = 16*)	0.81	0.53–0.93	0.66

*r*; Pearson′s correlation coefficient, 95% CI; 95% confidence interval calculated using the Fisher′s transformation, R^2^; coefficient of determination

**Fig 2:**
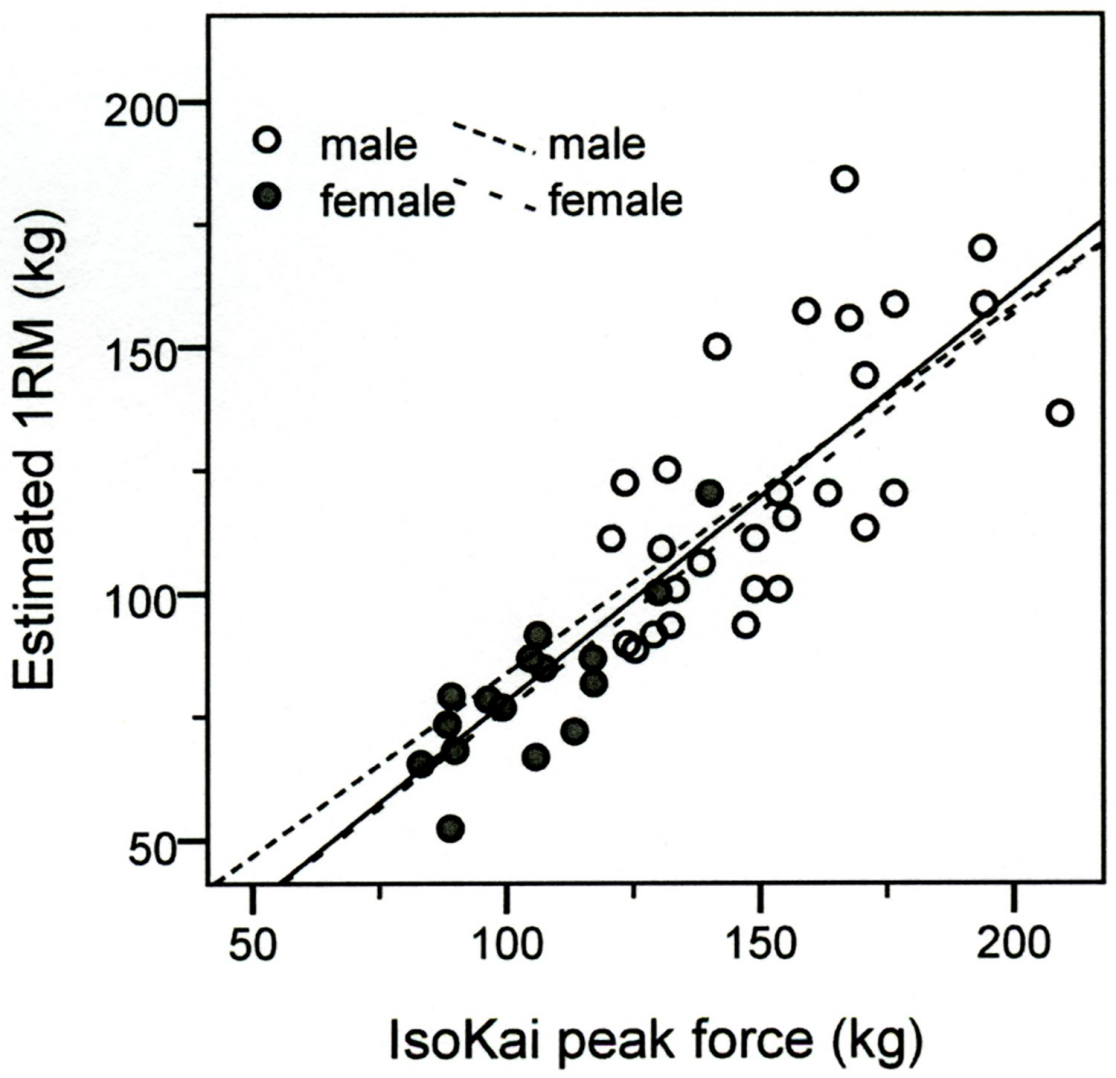
Scatterplot with linear regression lines for the correlation between the IsoKai peak force (IsoKai_Peak_) and the estimated 1RM (RM_est_) deadlift weights. Black line; total study sample, Hatched black lines; males and females separately.

The scatterplot including linear regression lines for the correlation between the IsoKai_Peak_ (kg) and the 1RM_est_ (kg) from the 5-10RM_DL_ tests is illustrated in Fig 2.

The paired t-test showed that the means of IsoKai_Peak_ and the means of 1RM_est_ differed by 20% in the total sample and in males, and by 23% in females (Table 3).

**Table 3:** Means of the IsoKai_Peak_ and the associated estimated 1RM (1RM_est_) deadlift weights.

	IsoKai_Peak_ kg (SD)	1RM_est_ kg (SD)	Diff kg (SD)	Diff %
All (*n = 44*)	136 (32)	108 (31)	27* (18)	20
Males (*n = 28*)	153 (24)	123 (27)	30* (21)	20
Females (*n = 16*)	105 (16)	80 (16)	24* (10)	23

Diff; difference between the IsoKai_Peak_ and the 1RM_est_ weight derived from the 5-10RM_DL_ tests, SD; standard deviation.

*significant difference (p<0.001).

In the univariate analyses, factors sex, weight, height and BMI, met the criteria to be included in the multivariable analysis. Weight and height showed collinearity with both sex and BMI. We considered the most logical alternative to avoid collinearity and therefore weight and height were excluded from further analyses. This resulted in a multivariable linear regression with the 1RM_est_ as dependent factor, and IsoKai_Peak_, sex and BMI as independent factors. During the sequential backward selection procedure, sex was excluded, resulting in a final linear regression for calculating the 1RM_est_ deadlift weight (kg) using the IsoKai_Peak_ (N) presented in Table 4.

**Table 4:** Final linear regression estimates from the multivariable analysis for converting the IsoKai_Peak_ (N) to an estimated 1RM (1RM_est_) deadlift weight (kg), *n = 44*.

1RM_est_ (kg)	β	SE	95% CI	B
Intercept	-51.63	24.32		
IsoKai_Peak_ (N)	0.08	0.01	0.06–0.09	0.76
BMI (kg/m^2^)	2.28	1.04	0.17–4.39	0.19

β; beta-coefficient, SE; standard error, 95% CI; 95% confidence intervals, B; standardised beta-coefficient.

The final regression had an adjusted R^2^ of 0.72 and SEE of 16.57 kg. Based on the final regression, an equation for converting the IsoKai_Peak_ to a 1RM_est_ deadlift weight would be; 1RM_est_ deadlift weight (kg) = -51.63 + (0.08 x IsoKai_Peak_) + (2.28 x BMI). As an example, a person with an IsoKai_Peak_ of 1330 N and a BMI of 25 kg/m^2^, as corresponding to the mean values for the total sample in this study, would get a 1RM_est_ deadlift weight of; -51.63 + (0.08 x 1330) + (2.28 x 25) = 111.77 kg.

## Discussion

In this first study to assess the concurrent validity of the IsoKai isokinetic lift test, the test demonstrated a good-to-excellent level of correlation with a submaximal 5-10RM deadlift test. The result showed that the IsoKai_Peak_ is a valid measure of maximal dynamic lifting capacity in a sample of SwAF employees.

The two lift tests in this study (the IsoKai_lift_ and the 5-10RM_DL_) are both demanding with respect to lifting technique [1]. The association of muscle strength provided by these two tests could be affected by the type and speed of muscle contraction, and because previous experience of the two tests differed between participants. Nevertheless, we found a 70 percent explained variance between the 1RM weight estimated (1RM_est_) by the 5-10RM_DL_ test and the IsoKai_peak_.

A comparison between the IsoKai_peak_, directly converted from Newton to kg, and the 1RM_est_ deadlift weights in kg, showed the 1RM_est_ deadlift weights to be, on average, 20% lower than IsoKai_Peak_ measures. This systematic difference was expected as we compared two different test modalities, but the amplitude has not been established previously. The differences found may be used as a rough estimate of what the IsoKai_peak_ means in actual lifting capacity in kg. But as an attempt to find a more accurate way of conversion, we developed an equation for calculating a 1RM_est_ deadlift weight in kg using the IsoKai_peak_ in Newton. The equation explained 72% of the total variance in the 1RM_est_ deadlift weight and had a SEE of 16.57 kg. The explained variance indicates that the equation could be useful for converting the IsoKai_peak_ in Newton to a 1RM_est_ deadlift weight on group level, e.g. in interventions targeting lifting capacity. However, the amplitude of the SEE corresponding to about 15% of the mean 1RM_est_ of 108 kg in the total sample implied a moderate accuracy when using the equation in individuals.

We were unable to find any study concerning validity of isokinetic lift tests in relation to deadlift. However, four studies investigated the concurrent validity between isokinetic lift tests and measures of maximal box lifting capacity [20–23]. Pytel et al. compared an isokinetic lift test, using the peak force as measure, with a maximum dynamic box lift in 10 male and 10 female college students and found, similar to the present study, good-to-excellent correlation with lower correlation coefficients for males (0.87) than for females (0.92) [20]. The same group repeated the study design with data from 48 male steel mill workers where a correlation of 0.47 was found between the two lift procedures [21]. Jacobs et al. detected a correlation of 0.96 when comparing the relationship between an isokinetic lift test mean peak force measure and operational box lifting test in 22 male and 28 female students [22]. In contrast to our results and the results by Pytel et al., they reported higher correlation in males (r = 0.88) than in females (r = 0.79). Within a population of 19 male and 6 female college students, Mital et al. revealed a correlation of 0.52 between an averaged force of three isokinetic lifts and maximum box lifting capacity [23]. We believe that these study results support our finding that an isokinetic lift test could be regarded as a valid test of maximal dynamic muscular strength. Similar to our study, Pytel et al. and Jacobs et al. used linear regression analyses to construct equations for calculating maximal lifting capacity based on the isokinetic force measure [20, 22]. In the equations, Pytel et al. included sex as an independent factor in addition to the isokinetic force measure, whereas Jacobs et al. included sex and body weight with the aim of increasing the accuracy of the estimated maximal lifting capacity. Likewise, we considered these factors in the development of our equation, but found BMI to be the factor that, together with the IsoKai_Peak_, best predicted the maximal lifting capacity expressed as the 1RM_est_ deadlift weight.

Based on the ‘a priori’ power calculation performed in this study, we believe our sample to be of appropriate size when analysing the total sample, but may be regarded as small for the evaluations on males and females. The statistical methods used in the development of the equation followed recommendations by Vittinghoff et al., with the equation derived in two phases in order minimise the risk of over-dispersion, and by using conservative p-values to minimize the risk of type II errors [19].

Though the use of the 5-10RM_DL_ to calculate a 1RM_est_ weight may be regarded as a limitation, this approach is an accepted practice intended to decrease the risk of injury. We also acknowledge that the Mayhew equation we used was developed for predicting the 1RM bench press weight. However, LeSuer et al. found a correlation of 0.95 between predicted and observed 1RM using the Mayhew equation for deadlift. They also found the Mayhew equation to underestimate the predicted 1RM deadlift weight with 10%, something we adjusted for in our analyses [17].

The variability in the data increased with larger strength measures indicating poorer accuracy with larger values (Fig 2). A possible explanation could be that larger values allow for larger differences than smaller values. However, these large differences were mainly seen in two of the male participants. Because of this, we reanalysed the data without these two male participants, as well as with each of them separately excluded (results not shown). These analyses resulted in correlations ranging from 0.85 to 0.86 in the total sample, which did not change the overall interpretation of our results. Though these results demonstrate validity, validity of a measurement applies only to the population in which it was tested why further validation of the IsoKai isokinetic lift is required in other populations where it is intended to be used.

Decreased accuracy of a 1RM_est_ deadlift weight calculated from a 5-10RM_DL_, the heterogeneity of the study population, and the fact that lifting is a multi-joint movement may explain the large variation of 15% (SEE = 16.57 kg) in the derived equation. A measurement error inherent to the device itself, since the IsoKai_Peak_ is registered momentarily, or differences in the force produced by the participant at that specific moment, may also contribute to less accuracy and larger variation.

Physically demanding work tasks are common in military settings, with lifting being one of the most frequent work tasks as well as being a strong risk factor for musculoskeletal injuries [24–26]. In a scientific report from Karolinska Institutet (2011), Swedish recruits estimated their heaviest loads lifted during combat training to be over 125 kg, with median loads estimated to be 60 kg, for males, and 35 kg for females. Loads lifted differed across different assignments, with male ranger recruits reporting lifting the heaviest loads followed by engineer and artillery recruits. Heavy objects lifted were, for example, personal equipment, boxes, grenades, tents and electric generators. In a thesis from 2009, Swedish ranger conscripts were found to lift and carrying backpacks weighing up to 55 kg [27]. Loads lifted and carried by US soldiers and UK soldiers range from 4.5 to 85 kg per person and 10 to 110 kg per person, respectively [28]. Considering this, valid muscular strength test assessing lifting capacity is of great importance in military settings.

The strong association found between the IsoKai_lift_ test and the 5-10RM_DL_ test, and the previously validated content validity of the IsoKai_lift_ for lifting and digging, supports the use of the test as an admission test for military personnel [10]. The equation derived in this study may increase the accuracy of assigning personnel to work duties in the SwAF based on their strength capabilities. The equation could also be used to calculate the intensity of strength training protocols, however it needs to be cross-validated in an independent sample before use in real practice.

## Conclusion

The IsoKai_lift_ test showed good-to-excellent correlation with the submaximal 5-10RM_DL_ test, and could thus be regarded as a valid measure of maximal dynamic muscular strength related to lifting capacity in SwAF personnel. Though this study was limited to a SwAF population, the results along with substantial supporting literature indicate that the IsoKai_lift_ test could be considered a valid test for muscular strength in other healthy adult populations such as military, police, firefighters and athletes. The derived equation could be used for converting the IsoKai_lift_ test peak force (N) to a 1RM_est_ deadlift weight (kg). However, the large variation associated with the 1RM_est_ deadlift weight is important to consider.

## Supporting information

S1 Appendix Leisure physical activity and physical work load. (DOCX)

S1 Dataset (XLSX)
