# Supplementary material for: Concurrent validity of an isokinetic lift test used for admission to the Swedish Armed Forces
Source: PLoS One. 2018 Nov 6;13(11):e0207054. doi: 10.1371/journal.pone.0207054 (PMC6219807; doi:10.1371/journal.pone.0207054)
Supplement: S1 Appendix — (DOCX) [file pone.0207054.s001.docx]

## S1 Appendix. Leisure physical activity and physical work load.

The question regarding leisure physical activity and physical work load.
Reference; Ekblom-Bak et al. (2011) [1].
The original Swedish version (below) has freely been translated to English and further categorised by the authors of this study.

**Leisure physical activity:**

**How much do you have in general moved or exerted yourself physically in your leisure time during the past year? NOTE! Please check all that apply to you.**

- - a) Very little
  - b) Very little but with sporadic walking or similar
  - c) Habitual physical activity such as gardening, walking or cycling to and from work, walking the dog, cleaning etc.
  - d) Have (in addition to activities in c), devoted me to easier forms of exercise like walking (or other activities with equivalent effort) at least once a week.
  - e) Have devoted me to more strenuous exercise such as fast walking, jogging, swimming, gymnastics or the equivalent at least once a week.
  - f) Have regularly devoted myself to hard training or competition where physical exertion has been great, such as running, different ball games or strength training.

In this study further categorised as:

- Low leisure physical activity; a), b) and c)
- Moderate leisure physical activity; d)
- High leisure physical activity; e)
- Very high leisure physical activity; f)

**Physical work load:**

**How physically stressful have your daily work or your daily pursuit been in the past year?**

- - a) Predominantly sedentary = Sedentary
  - b) Easy physical work but there I am moving a lot = Low physical work load
  - c) Pretty physically strenuous work = Moderate physical work load
  - d) Much physically strenuous work = High physical work load

**Original Swedish version:**

**Hur mycket har du i allmänhet rört dig eller ansträngt dig kroppsligen på din *fritid* under senaste året? OBS! Sätt kryss för *alla* alternativ som stämmer in på dig.**

- - a) Har rört mig mycket litet.
  - b) Har rört mig mycket litet men ibland tagit några enstaka promenader eller liknande.
  - c) Har fått ”vardagsmotion” i samband med städning, att gå i trappor, trädgårdsarbete, sällskapsdans, promenader eller lättare cykelturer till och från arbetet, gå ut med hunden etc.
  - d) Har (utöver aktiviteter i c), ägnat mig åt lättare forma av motion som promenader (eller andra aktiviteter med motsvarande ansträngning) ***minst en gång per vecka.***
  - e) Har ägnat mig åt mer anstängande motion som t ex snabba promenader, jogging, simning, motionsgymnastik eller motsvarande ***minst en gång per vecka.***
  - f) Har regelbundet ägnat mig åt ***hård träning eller tävling*** där den fysiska ansträngningen varit stor, t ex löpning, olika bollspel eller styrketräning.

**Hur fysiskt ansträngande har ditt dagliga arbete eller din dagliga sysselsättning varit under det senaste året?**

- - a) Övervägande stillasittande.
  - b) Lätt fysiskt arbete men där jag rör mig en hel del.
  - c) Ganska fysiskt ansträngande arbete.
  - d) Mycket fysiskt ansträngande arbete.

1. Ekblom-Bak E, Engström L-M, Ekblom B, Ekblom Ö. LIV 2000. Stockholm: 2011 2011-02-18. Report No.: 1.
